# Supplementary material for: The development status and future trends of lubricant additives technology: Based on patents analysis
Source: PLoS One. 2024 Jun 3;19(6):e0304888. doi: 10.1371/journal.pone.0304888 (PMC11146714; doi:10.1371/journal.pone.0304888)
Supplement: S1 Table — (DOCX) [file pone.0304888.s001.docx]

# Supplementary information

**S1 Table**

**Patent Analysis of Lubricant Additives Technolgy: Status and** **Future Trends**

Mianqing Wang^1^, Hua He^2^, Xi Fang^3^, Hui Li^4*^

1. School of Intellectual Property, Shanghai University, Shanghai 200444, Shanghai, China
2. School of Management, Shanghai University, Shanghai 200444, Shanghai, China
3. School of China-Europa Intellectual Property, Shanghai Institute of Technology University, Shanghai 201418, Shanghai, China
4. Office of Scientific Research, Shanghai Technical Institute of Electronics&Information University, Shanghai 201411, China;

*Corresponding author: Hui Li, Office of Scientific Research, Shanghai Technical Institute of Electronics&Information University, No.910, Jianchuan Road, Minhang District, Shanghai 201411, Shanghai, PR China

Tel.: +86 021 57134786; fax: +86 021 57131138.

1. mail address: 865100682@qq.com (Hui Li)

**S1 Table. Types of Lubricant Additives and Their Functional Properties**

| Types | Products | Functional Properties |
| --- | --- | --- |
| Single agent | Detergents | Used in the formulation of internal combustion engine oils, marine oils, metalworking oils, etc. |
|  | Dispersant | Used to formulate internal combustion engine oil, also can be used for preparing petrochemical auxiliaries and emulsified explosives, etc. |
|  | Antioxidant | Used to formulate internal combustion engine oil, gear oils, metal working oils, hydraulic oils, etc. |
|  | Viscosity Index Improver | Used to formulate internal combustion engine oils, gear oils, hydraulic fluids, etc. |
|  | Pour Point Depressant | Used to formulate internal combustion engine oils, gear oils, hydraulic fluids, etc. |
|  | Antirust Additive | Used to formulate internal combustion engine oils, transmission fluids, gear oils, hydraulic oils, metal working oils and greases, etc. |
|  | Anti-Wear Agent | Used to formulate gear oils, hydraulic fluids, metalworking fluids, hydraulic oils, etc. |
|  | Friction Modifier | Used to formulate gear oils, hydraulic fluids, metalworking fluids, hydraulic oils, etc. |
|  | Anti-Foam Additive | Used to formulate gear oils, hydraulic fluids, metalworking fluids, hydraulic oils, etc. |
| Compound agent | Internal Combustion Engine Oil Compound Agent | Used to formulate internal combustion engine oils. |
|  | Gear Oil Compound Agent | Used to formulate gear oils. |
|  | Hydraulic Fluid Compound Agent | Used to formulate hydraulic fluids. |
|  | Metalworking Oil Compound Agent | Used to formulate metalworking cooling lubricants |
|  | Antioxidant and Antirust Hydraulic Oil Compound Agent | Industrial Oil Compounds |
|  | Anti-Wear Hydraulic Fluid Compound Agent | Industrial Oil Compounds |
|  | Ashless High-Pressure Anti-Wear Hydraulic Oil Compound Agent | Industrial Oil Compounds |
|  | Hydraulic Guide Oil Compound Agent | Industrial Oil Compounds |
|  | Guide Oil Compound Agent | Industrial Oil Compounds |
|  | High-Temperature Chain Oil Compound Agent | Industrial Oil Compounds |
|  | Turbine Oil Compound Agent | Industrial Oil Compounds |
|  | Compressor Oil Compound Agent | Industrial Oil Compounds |
|  | Heat Transfer Oil Compound Agent | Industrial Oil Compounds |
|  | Quench Oil Compound Agent | Industrial Oil Compounds |
|  | Transformer Oil Compound Agent | Industrial Oil Compounds |
