# Supplementary material for: The development status and future trends of lubricant additives technology: Based on patents analysis
Source: PLoS One. 2024 Jun 3;19(6):e0304888. doi: 10.1371/journal.pone.0304888 (PMC11146714; doi:10.1371/journal.pone.0304888)
Supplement: S2 Table — (DOCX) [file pone.0304888.s002.docx]

# Supplementary information

**S2 Table**

**Patent Analysis of Lubricant Additives Technolgy: Status and** **Future Trends**

Mianqing Wang^1^, Hua He^2^, Xi Fang^3^, Hui Li^4*^

1. School of Intellectual Property, Shanghai University, Shanghai 200444, Shanghai, China
2. School of Management, Shanghai University, Shanghai 200444, Shanghai, China
3. School of China-Europa Intellectual Property, Shanghai Institute of Technology University, Shanghai 201418, Shanghai, China
4. Office of Scientific Research, Shanghai Technical Institute of Electronics&Information University, Shanghai 201411, China;

*Corresponding author: Hui Li, Office of Scientific Research, Shanghai Technical Institute of Electronics&Information University, No.910, Jianchuan Road, Minhang District, Shanghai 201411, Shanghai, PR China

Tel.: +86 021 57134786; fax: +86 021 57131138.

1. mail address: 865100682@qq.com (Hui Li)

**S2 Table. Retrieval formula and results of patents**

| Code | Retrieval formula | Results |
| --- | --- | --- |
| S1 | (TACD_ALL:(“Lubricant Additives”) OR TACD_ALL:(“Lubricating oil additive”) OR TACD_ALL:(“grease additive”) ) | 81289 |
| S2 | (TACD_ALL:(“Lubricant Additives”) OR TACD_ALL:(“Lubricating oil additive”) OR TACD_ALL:(“grease additive”) ) AND TACD_ALL:(“ditergent” OR "dispersant“ ) | 33848 |
| S3 | (TACD_ALL:(“Lubricant Additives”) OR TACD_ALL:(“Lubricating oil additive”) OR TACD_ALL:(“grease additive”) ) AND TACD_ALL: ( anti-oxidation AND corrosion additive) | 2781 |
| S4 | (TACD_ALL:(“Lubricant Additives”) OR TACD_ALL:(“Lubricating oil additive”) OR TACD_ALL:(“grease additive”) ) AND TACD_ALL: ( "extreme pressure additive" ) | 11140 |
| S5 | (TACD_ALL:(“Lubricant Additives”) OR TACD_ALL:(“Lubricating oil additive”) OR TACD_ALL:(“grease additive”)) AND TACD_ALL: ("oiliness additive" OR "friction modifier" ) | 23747 |
| S6 | (TACD_ALL:(“Lubricant Additives”) OR TACD_ALL:(“Lubricating oil additive”) OR TACD_ALL:(“grease additive”) ) AND TACD_ALL:("viscosity index improver" ) | 14953 |
| S7 | (TACD_ALL:(“Lubricant Additives”) OR TACD_ALL:(“Lubricating oil additive”) OR TACD_ALL:(“grease additive”)) AND TACD_ALL:(" antirust additive" ) | 1735 |
| S8 | (TACD_ALL:(“Lubricant Additives”) OR TACD_ALL:(“Lubricating oil additive”) OR TACD_ALL:(“grease additive”)) AND TACD_ALL:("pour point depressant " ) | 14857 |
| S9 | (TACD_ALL:(“Lubricant Additives”) OR TACD_ALL:(“Lubricating oil additive”) OR TACD_ALL:(“grease additive”) ) AND TACD_ALL:( “anti-foam additive” ) | 577 |
| S10 | (TACD_ALL:(“Lubricant Additives”) OR TACD_ALL:(“Lubricating oil additive”) OR TACD_ALL:(“grease additive”) ) AND TACD_ALL:( anti-emulsifier additive) | 384 |

**S3 Table. IPC codes related to Lubricant additive and number of Top 10 records**

| Number | IPC codes | Records |
| --- | --- | --- |
| 1 | C10N30: Specified physical or chemical property which is improved by the additive characterising the lubricating composition, e.g. multifunctional additives | 24,838 |
| 2 | C10N40: Specified use or application for which the lubricating composition is intended | 19,985 |
| 3 | C10M169: Lubricating compositions characterised by containing as components a mixture of at least two types of ingredient selected from base-materials, thickeners or additives, covered by the preceding groups, each of these compounds being essential | 13,032 |
| 4 | C10M159: Lubricating compositions characterised by the additive being of unknown or incompletely defined constitution (carboxylic acids with less than 30 carbon atoms in the chain, of unknown or incompletely defined constitution C10M 129/56) | 11,865 |
| 5 | C10M133: Lubricating compositions characterised by the additive being an organic non-macromolecular compound containing nitrogen | 10,722 |
| 6 | C10M129: Lubricating compositions characterised by the additive being an organic non-macromolecular compound containing oxygen | 8,397 |
| 7 | C10L1:Liquid carbonaceous fuels | 8,050 |
| 8 | C10M135: Lubricating compositions characterised by the additive being an organic non-macromolecular compound containing sulfur, selenium or tellurium | 7,922 |
| 9 | C10N20: Specified physical properties of component of lubricating compositions | 7,896 |
| 10 | C10N10: Metal present as such or in compounds | 7,629 |
