# Supplementary material for: The development status and future trends of lubricant additives technology: Based on patents analysis
Source: PLoS One. 2024 Jun 3;19(6):e0304888. doi: 10.1371/journal.pone.0304888 (PMC11146714; doi:10.1371/journal.pone.0304888)
Supplement: Attachment — Submitted filename: Response to Reviewers(2).docx [file pone.0304888.s004.docx]

**A point-by-point response to Journal requirement and reviewers’ comments**

Ref：PONE-D-23-35055

Tittle: The development status and future trends of lubricant additives technology: based on patents analysis

We sincerely thank the editors and reviewers for their careful reading of our manuscript and constructive comments on our work. In the following, our point-by-point response to each comment is highlighted in blue. We have revised the manuscript (highlighted in red) and Supporting Information accordingly.

Journal requirement:

1. When submitting your revision, we need you to address these additional requirements.

**Response:**

Thanks for yor reminder, we have update the text as suggested.

It is notable that I can’t open your second link “[https://journals.plos.org/plosone/s/file?id=ba62/PLOSOne_formatting_sample_title_authors_affiliations.pdf.](https://journals.plos.org/plosone/s/file?id=ba62/PLOSOne_formatting_sample_title_authors_affiliations.pdf.)”, so I don’t know whether I fully meet the requirement of style format.

2. Note from Emily Chenette, Editor in Chief of PLOS ONE, and Iain Hrynaszkiewicz, Director of Open Research Solutions at PLOS: Did you know that depositing data in a repository is associated with up to a 25% citation advantage ([https://doi.org/10.1371/journal.pone.0230416](https://doi.org/10.1371/journal.pone.0230416))? If you’ve not already done so, consider depositing your raw data in a repository to ensure your work is read, appreciated and cited by the largest possible audience. You’ll also earn an Accessible Data icon on your published paper if you deposit your data in any participating repository ([https://plos.org/open-science/open-data/#accessible-data](https://plos.org/open-science/open-data/#accessible-data)).

Thanks for your reminder, as mentioned in our article, all of our data comes from the Patsnap patent database, which has the power to allow us to perform patent analysis directly on the platform without downloading the data. After all, 77,701 patents are a large amount of data, which is difficult to download. In order to meet the need for open access, we still share some of the data used for analysis in this article to Dryad for researchers' needs. The accessible Data link is DOI: 10.5061/dryad.k6djh9wf7

3. We note that Figure 1 in your submission contain [map/satellite] images which may be copyrighted. All PLOS content is published under the Creative Commons Attribution License (CC BY 4.0), which means that the manuscript, images, and Supporting Information files will be freely available online, and any third party is permitted to access, download, copy, distribute, and use these materials in any way, even commercially, with proper attribution. For these reasons, we cannot publish previously copyrighted maps or satellite images created using proprietary data, such as Google software (Google Maps, Street View, and Earth). For more information, see our copyright guidelines: [http://journals.plos.org/plosone/s/licenses-and-copyright.](http://journals.plos.org/plosone/s/licenses-and-copyright.)

**Response:**

Thank you for your kind reminder and sorry for our oversight. In order to avoid copyright issues, we have followed your suggestion and removed the original Figure 1 and used new Figures 1 and 2 to supplement our study (which in has highlighted in red in paper). Specifically, we divided the original figure 1 into new figure 1 Spatial distribution of patents application and new figure 2 Temporal distribution of patents application, as follow:

|  | Counts |
| --- | --- |
| China | 12544 |
| American | 11838 |
| Japan | 10797 |
| EPO | 8005 |
| Germany | 5220 |
| WIPO | 4240 |
| Canada | 3524 |
| Australia | 2765 |
| Korea | 2228 |
| singapore | 1025 |
| UK | 1096 |
| others | 3684 |
| France | 1454 |
| India | 1235 |
| Brazil | 1227 |
| Austria | 1196 |
| Russia | 1062 |
| Norway | 791 |
| Spain | 786 |
| Mexico | 707 |
| Netherlands | 696 |
| Poland | 471 |
| Finland | 359 |
| Belgium | 329 |
| South Africa | 277 |
| Italy | 145 |

Figure 1 Spatial distribution of patents application

Numbers of Patents

|  | France | Australia | Korea | Canada | WIPO | Germany | EPO | Japan | China | American |
| --- | --- | --- | --- | --- | --- | --- | --- | --- | --- | --- |
| 1965 | 30 | 15 | 0 | 0 | 0 | 72 | 0 | 0 | 0 | 81 |
| 1966 | 34 | 18 | 0 | 0 | 0 | 93 | 0 | 0 | 0 | 91 |
| 1967 | 19 | 8 | 0 | 0 | 0 | 65 | 0 | 0 | 0 | 79 |
| 1968 | 22 | 13 | 0 | 1 | 0 | 107 | 0 | 0 | 0 | 78 |
| 1969 | 78 | 21 | 0 | 1 | 0 | 92 | 0 | 2 | 0 | 79 |
| 1970 | 64 | 16 | 0 | 1 | 0 | 77 | 0 | 0 | 0 | 77 |
| 1971 | 63 | 8 | 0 | 1 | 0 | 93 | 0 | 1 | 0 | 105 |
| 1972 | 78 | 23 | 0 | 8 | 0 | 106 | 0 | 11 | 0 | 87 |
| 1973 | 41 | 24 | 0 | 7 | 0 | 77 | 0 | 8 | 0 | 82 |
| 1974 | 44 | 18 | 0 | 15 | 0 | 80 | 0 | 26 | 0 | 100 |
| 1975 | 51 | 11 | 0 | 28 | 0 | 80 | 0 | 48 | 0 | 89 |
| 1976 | 43 | 28 | 0 | 25 | 0 | 86 | 0 | 56 | 0 | 89 |
| 1977 | 67 | 22 | 0 | 23 | 0 | 98 | 0 | 118 | 0 | 120 |
| 1978 | 90 | 24 | 1 | 34 | 1 | 70 | 20 | 127 | 0 | 129 |
| 1979 | 32 | 22 | 2 | 31 | 2 | 62 | 30 | 105 | 0 | 111 |
| 1980 | 44 | 31 | 7 | 28 | 3 | 59 | 41 | 117 | 0 | 93 |
| 1981 | 19 | 17 | 1 | 25 | 3 | 25 | 65 | 157 | 0 | 84 |
| 1982 | 40 | 24 | 1 | 28 | 2 | 55 | 69 | 198 | 0 | 77 |
| 1983 | 20 | 20 | 2 | 42 | 0 | 45 | 126 | 254 | 0 | 74 |
| 1984 | 17 | 26 | 4 | 31 | 7 | 34 | 127 | 278 | 0 | 98 |
| 1985 | 5 | 47 | 2 | 59 | 13 | 29 | 123 | 261 | 9 | 123 |
| 1986 | 8 | 60 | 9 | 77 | 22 | 73 | 181 | 329 | 28 | 126 |
| 1987 | 15 | 54 | 14 | 65 | 20 | 85 | 203 | 334 | 30 | 113 |
| 1988 | 7 | 72 | 21 | 61 | 22 | 91 | 210 | 363 | 26 | 147 |
| 1989 | 21 | 83 | 27 | 74 | 29 | 106 | 204 | 375 | 32 | 158 |
| 1990 | 0 | 74 | 24 | 109 | 25 | 118 | 251 | 373 | 22 | 172 |
| 1991 | 8 | 60 | 31 | 93 | 39 | 87 | 189 | 276 | 24 | 130 |
| 1992 | 5 | 61 | 19 | 70 | 42 | 96 | 158 | 192 | 27 | 147 |
| 1993 | 11 | 72 | 35 | 97 | 57 | 92 | 189 | 186 | 36 | 163 |
| 1994 | 5 | 90 | 23 | 83 | 53 | 77 | 182 | 172 | 43 | 144 |
| 1995 | 10 | 88 | 20 | 82 | 68 | 144 | 216 | 206 | 70 | 176 |
| 1996 | 8 | 122 | 41 | 114 | 76 | 177 | 249 | 248 | 86 | 152 |
| 1997 | 0 | 102 | 76 | 104 | 81 | 107 | 208 | 232 | 76 | 198 |
| 1998 | 8 | 80 | 69 | 120 | 76 | 124 | 207 | 225 | 101 | 133 |
| 1999 | 2 | 86 | 39 | 86 | 79 | 95 | 155 | 202 | 73 | 117 |
| 2000 | 6 | 92 | 37 | 81 | 86 | 110 | 168 | 171 | 110 | 103 |
| 2001 | 3 | 79 | 38 | 71 | 107 | 79 | 130 | 180 | 125 | 216 |
| 2002 | 4 | 102 | 34 | 74 | 112 | 88 | 143 | 165 | 124 | 245 |
| 2003 | 0 | 127 | 57 | 116 | 128 | 97 | 214 | 229 | 149 | 315 |
| 2004 | 5 | 62 | 67 | 109 | 145 | 87 | 210 | 264 | 206 | 245 |
| 2005 | 5 | 59 | 97 | 123 | 149 | 111 | 231 | 257 | 269 | 323 |
| 2006 | 7 | 81 | 120 | 146 | 217 | 116 | 313 | 381 | 315 | 362 |
| 2007 | 18 | 31 | 73 | 116 | 188 | 117 | 264 | 257 | 280 | 307 |
| 2008 | 24 | 21 | 94 | 94 | 151 | 83 | 199 | 297 | 302 | 290 |
| 2009 | 10 | 31 | 78 | 91 | 151 | 98 | 238 | 248 | 385 | 273 |
| 2010 | 15 | 34 | 98 | 88 | 145 | 87 | 229 | 313 | 438 | 262 |
| 2011 | 14 | 22 | 85 | 79 | 163 | 89 | 247 | 240 | 500 | 316 |
| 2012 | 7 | 45 | 108 | 100 | 175 | 88 | 243 | 268 | 557 | 317 |
| 2013 | 6 | 51 | 103 | 100 | 191 | 108 | 267 | 210 | 794 | 383 |
| 2014 | 9 | 35 | 123 | 66 | 150 | 88 | 230 | 281 | 749 | 274 |
| 2015 | 10 | 57 | 85 | 80 | 145 | 75 | 200 | 239 | 946 | 332 |
| 2016 | 8 | 35 | 77 | 63 | 141 | 67 | 200 | 269 | 857 | 335 |
| 2017 | 20 | 62 | 104 | 73 | 190 | 87 | 266 | 323 | 953 | 386 |
| 2018 | 14 | 23 | 76 | 56 | 207 | 59 | 207 | 228 | 944 | 384 |
| 2019 | 13 | 26 | 80 | 49 | 179 | 46 | 179 | 222 | 840 | 300 |
| 2020 | 3 | 17 | 78 | 49 | 147 | 23 | 144 | 138 | 816 | 273 |
| 2021 | 2 | 13 | 39 | 40 | 141 | 16 | 76 | 92 | 675 | 151 |
| 2022 | 0 | 0 | 9 | 5 | 92 | 2 | 4 | 45 | 474 | 50 |
| 2023 | 0 | 0 | 0 | 0 | 1 | 0 | 0 | 0 | 53 | 2 |

Figure 2 Temporal distribution of patents application

These two figures respectively show the time and spatial distribution of lubricant additive technology patent applications, which can achieve the effect of the original Figure 1.

**Reviewer: 1**

Comments:

The authors described “The development status and future trends of lubricant additives technology: based on patents analysis”. There are several major as well as minor mistakes in the manuscript. The authors are advised to check all the gaps and errors before its plausible publication in ‘PLOS ONE’.

**Response:**

We greatly appreciate the reviewer considering our work “is able to attract much attention of readers” and the nice summary of our paper.

1. How were the 77701 patents chosen? Were any specific filters applied to the Patsnap database, and if so, could these filters bias the results?

**Response:**

Thanks for kindly reminding us to clarify this point. Patsnap's filter was utilized in our study, however, it is important to note that these filters do not introduce bias into the results. Initially, our search formula yielded 81,845 patent applications (refer to Supplementary Material S2). The filters were employed to maintain strict control over our research data, focusing on patents submitted to the Patent Office between 1965 and 2023, thereby ensuring that our patent data fell within a reasonable time frame. Furthermore, we manually excluded certain patents that were unrelated to the field of lubricant additives(possibly removing dozens of patent applications), to minimize noise in the patent data. After this process, we finally get 77701 patent, we believe these adjustments did not impact the results of our study.

**We regret that we did not explain this clearly in the article and we have now re-explained this in the article. The revised content is as follows:**

Patsnap Patent Search and Analysis Database contains nearly 161 million patent data from nearly 126 countries/regions around the world, which is a rich and comprehensive patent database^[1]^. Additionally, its powerful patent analysis capability has garnered widespread favour among numerous universities and enterprises, serving as a widely adopted tool for patent analysis and intelligence gathering across various industries. Therefore, we conducted a patent search for lubricant additive technology through the Patsnap database. our data collection process mainly includes three steps:

Firstly, keywords such as 'lubricant' and 'lubricant additives' were used to retrieve patents, resulting in 81,845 patents. The specific Boolean search formula and search records is shown in Table S2 of the supplementary material.

Secondly, filters in the Patsnap database were applied to limit the patent data to the period between 1965 and 2023.

Furthermore, certain patents unrelated to lubricant additives were manually excluded to reduce noise in the dataset.

After this process, we ensure our dataset within a reasonable time frame (from 1965 to 2023), That is, all patent applications related to lubricant additive technology were submitted to the patent office between 1965 and 2023 in our paper. Finally, we obtained a total of 77701 patents and used Patsnap 's analysis capability to perform further analysis.

2. What specific methods were used to analyze both structured and unstructured data? Were there any limitations to these methods, and could they have affected the outcomes?

**Response:**

We thank the reviewer for giving us the opportunity to clarify this point.

In this study, the structured data of patent documents mainly includes information such as country of patent application, IPC code, and patent application time. We use statistical analysis, social network analysis, logistic models, and patent portfolio analysis to explore the current status, trends, hot spots, and potential of lubricant additive research. These methods can help us grasp the development status and future trends of lubricant additives at the macro level, but they may be somewhat insufficient for specific technologies at the micro level.

Unstructured data in patent documents mainly include titles and abstracts of patent documents. In this study, we used Patsnap's data mining technology to analyze the unstructured data of lubricant additive patents to identify the research hot spots in this field at the micro level. Specifically, we used the suffix tree clustering algorithm to cluster patent texts, ranked and visualized word frequencies, and obtained a keyword cloud diagram of lubricant additive patent technologies. The suffix tree clustering algorithm can fully utilize the approximate information between words to achieve ideal clustering effects. However, this algorithm requires a large number of data samples, and the text needs to be preprocessed, including word segmentation, stop word removal, stemming, and so on. The patent data of this study reached 77,701 pieces, which provided sufficient data samples for the algorithm. At the same time, Patsnap's data mining technology can effectively preprocess the text. Therefore, the structured data and unstructured data analysis methods used in this article will not affect the research results on the development status and trends of lubricant additives.

3. Was the Patsnap database chosen for specific reasons? Are there other databases that could have provided complementary information?

**Response:**

We thank the reviewer for giving us the opportunity to clarify this point.

As we mentioned in the article, Patsnap database records nearly 161 million patent data from nearly 126 countries/regions around the world, making it a rich and comprehensive patent database. Its powerful patent analysis capabilities have been widely supported by many universities and enterprises, and it has become a widely used patent analysis and intelligence tool in China. The database is updated every two weeks, which helps us capture relevant patent information in a timely and effective manner. With Patsnap's powerful patent analysis function, we can conduct patent analysis directly on the platform to meet our needs for structured and unstructured patent data analysis of lubricant additives. Of course, there are other patent databases with the same functions in China, such as Patentics, Innography, etc., as well as the international Derwent database. But we are more familiar with using Patsnap. Its powerful analysis functions and friendly interactive interface allow us to easily focus on researching the paper.

**In case you want to know more about it, we list some papers that use Patsnap to perform their patent analysis:**

[1] QIAO Z, YOU J, ZOU Y, et al. Analysis of the development trend of small nucleic acid drugs[J]. China Pharmacy, 2022: 1842-1847.
[2] Luan C, Sun X, Wang Y. Driving forces of solar energy technology innovation and evolution[J]. Journal of Cleaner Production, 2021, 287: 125019.
[3] Liu M, Guo J, Bi D. Comparison of administrative and regulatory green technologies development between China and the US based on patent analysis[J]. Data Science and Management, 2023, 6(1): 34-45.
[4] Liang D, Wang Y F. Analysis of low-fat food research and development patents by PatSnap[J]. Journal of Food Safety and Quality, 2019, 10(8): 2420-2424.
[5] Wang J. Patent information analysis of Anhui Agricultural University based on PatSnap[J]. 2021.
4. How were "countries with the largest number of patents" and "main exporting countries" quantified? Are there other ways to measure patent activity that could offer further insights?

**Response:**

Thanks for kindly reminding us to clarify this point,As outlined in our study, the term 'country with the largest number of patents' pertains to the nation that has filed the greatest number of patent applications for lubricant additives with the patent office between 1965 and 2023. China leads in this aspect with 12,544 patents. On the other hand, the term 'main exporting country' denotes the nation from which technical knowledge emanates, typically measured by the number of patent citations. The United States stands out as the primary source of knowledge outflow, with 29,108 citations of its lubricant additive patents by other countries. In addition, we can also examine patent activity related to lubricant additives, including trends in patent licensing, R&D teams, patentees, and highly cited patents. However, these aspects are not the primary focus of this study and are therefore not included in the article.

**We apologize for not making this clear in the article. Quantitative data has been added to our new version. The revised content is as follows:**

Figure 1 shows the global distribution of lubricant additive technology patent application numbers from 1965 to 2023.Regarding total patent applications, China leads with 12544 patent applications related to lubricant additives, indicating a strong demand in the market. Following closely are the United States (11838), Japan (10797), Germany (5220), Canada (3524), and France (1454), all representing developed industrial economies. This distribution underscores the substantial connection between lubricant additives and industrial economies.

As shown in Figure 3, The World Intellectual Property Organization (WIPO) and the European Patent Office (EPO) are intergovernmental organizations that facilitate the protection of intellectual property through collaboration among States and other international entities. They play a crucial role in connecting global knowledge networks. Technology exchanges are particularly robust among nations like China, the United States, Japan, Germany, France, Canada, the United Kingdom, and Russia. Notably, the United States leads in knowledge dissemination, with its lubricant additive patents being cited 29,108 times by other countries. In comparison, China's patents, despite their large number, received 4,924 citations (only 17% of the US), indicating the superior quality of US patents in this field. China, on the other hand, has the highest knowledge absorption globally, with 21,486 citations of foreign patents related to lubricant additives. This knowledge flow highlights China's reliance on external technical know-how to bridge its technology gap, explaining the rapid growth of its lubricant additives industry despite starting late..

5. How were "multifunctional composite additives" defined and identified? Are there other significant research areas that might have been overlooked?

**Response:**

Thanks to reviewer for this careful comment.

The 'multifunctional composite additives' are formulated by blending several single agents in specific ratios, resulting in a versatile agent with diverse properties. As shown in Table S1 of the supplementary material, these additives primarily include products like Internal Combustion Engine Oil Compound Agents, Gear Oil Compound Agents, Hydraulic Fluid Compound Agents, Metalworking Oil Compound Agents, and others. These products are widely utilized in industrial oil formulations. The identification of multifunctional compound additives can be approached from two main perspectives. Firstly, these additives can be identified based on the IPC code, as demonstrated in Table S3 of the supplementary materials. For instance, C10N30, C10M169, and C10M159 are categorized as multifunctional compound additives. Secondly, another method of identifying multifunctional compound additives is through analyzing the title and abstract of patent documents. In Chapter 3.3.2, the Keywords analysis of this paper utilizes data mining technology to conduct text mining on the title and abstract of patent documents, enabling the identification of multifunctional compound additives.

As to whether other significant research areas may have been overlooked. The main objective of this paper is to discuss and analyze the current status and development trend of lubricating oil additives. We have conducted sufficient industry background research before patent retrieval to ensure that we can obtain complete data, so we can be sure that almost all patent applications for lubricant additives are included in our data sample. However, it is important to note that while we have provided insights into the current landscape and future direction of lubricant additives, the specific technological aspects of these additives were not explored in depth. This limitation may result in overlooking the application of specific technologies within lubricant additives, as highlighted in the paper's limitations.

6. How were "environmentally friendly additive compositions" identified as a research hotspot? Are there other metrics that could be used to assess research trends?

**Response:**

We would like to thank the reviewer for this important comment.

This study utilizes data mining techniques to cluster patent application titles and abstracts related to lubricating oil additives. Word frequency analysis was conducted to identify the top 300~500 keywords for visualization, revealing the research focus of lubricant additives. The keyword cloud map (Figure 6) highlights keywords related to the composition and types of lubricating oil additives, as well as key ingredients like"fatty acids", "acrylic", "polyolefin", and others. These environmentally friendly polymers are versatile in meeting lubricating oil performance requirements across different products and environments, particularly in base oil thickening. Furthermore, high-frequency keywords such as "nanoparticl", "nanosheet", "microbial", and "biodegradable" suggest a growing interest in utilizing nanomaterials and degradable ingredients to enhance lubrication performance, reduce friction and wear under extreme conditions, and improve cost-effectiveness and environmental sustainability. The highlight of these keywords underscores the growing importance of"environmentally friendly additive compositions" in lubricant additive research.

In addition, combined with the analysis of the patent application trend of lubricating oil additives, IPC co-occurrence network, technology life cycle and the development potential of lubricating oil additives sub-technology in this paper, we can judge that the turning point of lubricating oil additive technology appeared in 2015, and it is currently in a relatively mature stage. The research and development of lubricating oil additives will be further combined with the current policies and regulations, industrial development and environmental protection needs, the traditional lubricating additives in sulfur, phosphorus, and nitrogen compounds are gradually replaced by non-toxic and odourless boron compounds, nano-compounds and so on. Moreover, the focus in technical fields such as C10M159, C10M135, C10M133, and C10M129 is on utilizing organic compounds to enhance the stability and durability of lubricating oil, showing strong development potential. Based on the above, we can also determine that the development of lubricant additives will be transferred to more economical and more environmentally friendly, application of nanomaterials, graphene and other degradable key components in the additive, to maintain lubrication performance under extreme conditions, to reduce friction and wear will be the focus of future innovation direction.

7. What specific criteria were used to determine the potential of "environmentally friendly and economically degradable additives"? Are there other factors that could influence their future success?

**Response:**

We thank the reviewer for this insightful comment.

This paper utilizes a significant amount of structured and unstructured data from patent applications related to lubricant additives to determine the potential of environmentally friendly and economically degradable additives. The specific criteria focus on two main aspects:

First, in conjunction with the technical life cycle of lubricating oil additives, it can be determined that the saturation point of lubricating oil additive technology was reached in 2015, leading to a slowdown in the growth rate of patent applications for lubricating oil additives. This indicates the maturity of traditional lubricating oil additive technology and the necessity to introduce breakthrough innovative technologies to meet current economic demands. Furthermore, considering the environmental initiatives of the Paris Agreement, global policies and regulations on environmental protection, growth trends in various IPC technical fields of lubricant additives, and current research hotspots, it is evident that additives focusing on environmental protection and economic degradability will be the future development direction of lubricating oil additives.

Additionally, through patent portfolio analysis, it is observed that multi-functional composite additives, such as non-toxic boron compounds and nano-compounds, are gaining widespread attention as replacements for traditional sulfur, phosphorus, and nitrogen compounds in lubricant additives. Technology areas like C10M159, C10M135, C10M133, C10M129, C10N40, and C10N20 are in early stages of development, showing significant prospects for sustainability. These trends suggest that the future focus of lubricant additives will prioritize sustainability, utilizing degradable components like nanomaterials and graphene to enhance lubrication performance, reduce friction and wear under extreme conditions, and improve cost-effectiveness and environmental friendliness

Certainlly, lubricating oil additives are called the barometer of industrial development. Thus, the growth of the industrial economy directly correlates with the demand for these additives. Additionally, environmental regulations, industry competition, technological advancements, and cost-effectiveness also play a significant role in shaping the future of environmentally friendly and economically degradable additives.

8. Does this study offer new insights into the lubricant additive field beyond what is already known? Does it contribute significantly to the understanding of research and application trends?

**Response:**

Thanks to reviewer for this careful comment.

This research provides valuable insights into lubricant additives that extend beyond current knowledge, contributing significantly to the understanding of research and application trends.

First, the study uncovers the present status and future directions in the lubricant additives field by analyzing a vast amount of patent data. Notably, the continuous growth in patent applications reflects the strong market demand for these additives. Moreover, the research identifies changing geographic distribution patterns and variations in IPC classification and research focus among different countries, offering a fresh outlook on the evolution of lubricant additive technology.

Second, the research focus and future development trend of lubricant additives are analyzed, highlighting the increasing attention towards multifunctional compound additives. The direction of future lubricant additive development is proposed to focus on sustainability, incorporating nanomaterials and other environmentally friendly ingredients. Additionally, the technology life cycle analysis indicates that the lubricating oil additives field reached saturation in 2015, with current technology being mature and patent application growth rates slowing down. This suggests the need for breakthrough innovations in traditional lubricating oil additive technology to align with current industrial needs. Sub-technology analysis reveals that while innovation activities in C10M159, C10M135, C10M133, C10M129, C10N40, and C10N20 are currently not very active, they hold significant development potential for the future.

These insights offer valuable guidance for future research and application of lubricant additive technology, steering the industry towards a more sustainable and environmentally friendly direction. This study provides fresh perspectives on lubricant additives and offers crucial insights into research and application trends. By shedding light on industry development dynamics and future directions, this study contributes to the advancement of lubricant additive technology and serves as a vital reference for research and practice in related fields.

9. Are the findings of this study applicable to different types of lubricants and industries? Are there limitations to the generalizability of the conclusions?

**Response:**

We thank the reviewer for giving us the opportunity to clarify this point

This study utilizes a comprehensive dataset of patent information, encompassing nearly all patent applications related to lubricant additives. As a result, the insights on the development status and trends of lubricant additives presented in this paper are broadly applicable across various lubricant types and industries. For instance, lubricant additives play a crucial role in sectors such as automotive, aerospace, manufacturing, and marine, where smooth equipment operation and friction reduction are paramount. The findings on additive trends, market dynamics, and future directions outlined in the study hold potential benefits for each of these industries.

However, it is important to note that the use of lubricant additives is intricately linked to industrial economic development, regulatory frameworks, and environmental factors specific to different regions. Consequently, the conclusions drawn in this study may have limitations in terms of industry-specific economic conditions, regulatory environments, and geographical variations.

Firstly, diverse industries may have distinct performance requirements, regulatory mandates, and market landscapes, which could impact the applicability of the study's insights to individual sectors. While the study's findings offer valuable guidance, they may not fully address the unique needs and challenges faced by each industry.

Secondly, while the research may reveal global trends, regional disparities in industrial practices, regulatory policies, and market preferences could restrict the generalizability of the conclusions to specific geographic areas.

Lastly, although the study focuses on lubricant additives, the efficacy and requirements of lubricants are influenced by a multitude of factors, including base oil quality, equipment design, and operational conditions. Therefore, to effectively implement the study's conclusions, other technical and operational considerations may need to be taken into account.

10. The authors are advised to mention the potential future research directions suggested by this study. Are there any specific areas that need further investigation?

**Response:**

We sincerely thanks you for your feedback which would help to improve the quality of our manuscript. We acknowledge our oversight in not providing suggestions for future research directions in the initial version of this paper. After carefully considering your feedback and synthesizing the research findings, we have revised the conclusion section to include recommendations for future research directions. **The revised content is as follows:**

Future research can be focused on various aspects of lubricating oil additives. Firstly, exploring the use of specific technologies such as sustainable lubricating oil additives, multifunctional compound additives, alternative additive compounds, and the integration of nanomaterials and graphene in additives from a microscopic perspective. Secondly, research on lubricating oil additives should consider the relationship with industrial economy, market competition, and industrial structure for a more comprehensive analysis. Lastly, further studies should delve into the specific mechanisms of lubricant additive technology, including the interactions between additives and base oils to understand their impact on reducing friction and wear. Addressing these research directions can advance the field of lubricant additives, enhancing performance, sustainability, and environmental compatibility of lubricant formulations.

11. How does the research and development of lubricant additives compare to other related fields, such as tribology and surface engineering?

**Response:**

Thanks for your valuable comments.

In the realm of mechanical engineering, the occurrence of friction and wear during mechanical motion has long been a hindrance to the longevity and effectiveness of mechanical systems. The utilization of lubricating oil additives presents a viable solution to this issue, effectively reducing energy loss resulting from friction and wear. Research in lubricant additives is primarily focused on enhancing the capabilities of lubricating oils to mitigate friction, wear, and corrosion. These additives, typically chemical compounds, work by creating a protective film on friction surfaces, thereby diminishing friction between mechanical components. Tribology, the scientific study of surface interactions encompassing friction, wear, and lubrication, delves into various aspects such as tribological principles, material characteristics, and surface contact mechanics. Scientists in the field of tribology strive to comprehend the fundamental principles governing surface interactions to anticipate the behavior of friction and wear in mechanical systems, as well as to devise novel lubrication techniques to optimize system performance. Surface engineering, on the other hand, is centered on altering surface properties to enhance performance and durability, often in conjunction with lubricants or lubricant additives. While lubricant additives are specifically tailored to enhance lubrication performance, tribology and surface engineering offer complementary strategies to enhance overall system efficiency and dependability through a more profound understanding of friction, wear, and surface interactions. By fostering collaboration and interdisciplinary approaches across these disciplines, significant strides can be made in the design and optimization of mechanical systems.

12. What are the biggest challenges facing the development of new and improved lubricant additives?

**Response:**

We would like to thank the reviewer for these constructive comments.

The development of new and improved lubricant additives faces significant challenges in various aspects. Compliance with Environmental Regulations is a key hurdle, as meeting strict criteria for biodegradability, toxicity, and environmental impact while maintaining or enhancing performance is challenging due to increasingly stringent regulations. Additionally, Meeting Performance Requirements poses a significant challenge, as developing additives that effectively reduce friction, wear, and heat generation, while also protecting against corrosion and oxidation, across various operating conditions and applications is complex. Ensuring Compatibility is another obstacle, as achieving stability, homogeneity, and effectiveness by ensuring compatibility with base oils and other additives can be difficult, especially when incorporating new chemistries or functionalities. Cost-effectiveness is a crucial factor, as balancing the cost of research, development, testing, and manufacturing against the potential benefits and market demand for improved additives is a challenge. Sustainability is also a key concern, as meeting the growing demand for sustainable additives derived from renewable raw materials and incorporating eco-friendly chemistries while optimizing manufacturing processes for sustainability presents challenges. Furthermore, challenges include Market Competition, Testing and Validation, and Keeping Pace with Technological Advances. Addressing these challenges necessitates collaboration among industry stakeholders to foster innovation, ensure compliance, and meet the evolving needs of industries and consumers.

Based on our research, the trend in patent applications, technical layout, technical development stages, and research hotspots indicates a shift towards utilizing degradable raw materials in the research and development of lubricant additives. The focus is on creating multi-functional composite additives with diverse properties, representing an important effort in addressing the challenges associated with developing new lubricant additives.

13. What are the ethical considerations associated with the use of lubricant additives?

**Response:**

We would like to thank the reviewer for this important comment.

The ethical considerations surrounding lubricant additives primarily involve environmental impact, health and safety, and sustainability. Lubricant additives, particularly those containing heavy metals or toxic substances, can have detrimental effects on the environment if not properly managed. When released into soil or water, these additives can pollute ecosystems, harm wildlife, and disrupt natural processes. In terms of health and safety, certain lubricant additives may pose risks to workers involved in their production, handling, or application, potentially leading to occupational diseases or injuries. Ethical practices in this area include implementing safety protocols, providing adequate training, and minimizing exposure to harmful additives. Additionally, ethical concerns extend to the sustainability of lubricant additives and their production processes. Businesses should aim to reduce resource consumption, minimize waste generation, and explore environmentally friendly alternatives. Sustainable practices, such as utilizing renewable raw materials, optimizing manufacturing processes, and promoting recycling or reuse, play a vital role in environmental protection and social responsibility.

In conclusion, the ethical use of lubricant additives necessitates a delicate balance between performance, safety, and sustainability, with a primary focus on the well-being of individuals, communities, and the environment. This study highlights that lubricating oil additives are progressing towards environmentally friendly and sustainable practices. Notably, patent applications for lubricating oil additives are increasingly focused on developing multifunctional composite agent products. Additionally, the use of 'fatty acids', 'acrylic', 'polyolefin', 'fatty alcohol', 'salicylate', and 'alpha olefin' as raw materials for lubricating oil additives showcases a shift towards utilizing green and health-conscious ingredients. These advancements aim to enhance the performance and sustainability of lubricating oil additives while minimizing their potential environmental and human health impacts.

14. There are so many typos and grammatical mistakes in the entire manuscript, that the authors are advised to rectify all these errors.

**Response:**

Thank you for bringing this matter to our attention. We have made additional revisions to the English writing to enhance clarity and correctness in this manuscript. The modifications include adjustments to spelling and language to align more closely with English writing conventions. These changes have been marked in red within the text.

We would like to thank you for your professional review work, constructive comments, and valuable suggestions on our manuscript. Please don’t hesitate to contact with me if you have any question, we wish we can work together to improve the quality of this paper.

1. https://www.patsnap.com/
